# Advances in Drug and Vaccine Delivery for Low- and Middle-Income Healthcare Programs—The Case for Replacing Multi-Dose Vials with Prefilled Single-Dose Delivery Systems

**DOI:** 10.3390/pharmacy13060180

**Published:** 2025-12-10

**Authors:** Darin Zehrung, Michael J. Free

**Affiliations:** 1 Independent Consultant, Stanwood, WA 98292, USA; 2 Independent Consultant, Clinton, WA 98236, USA; mobe@whidbey.net

**Keywords:** auto-disable, blow–fill–seal, immunization, infection control, injections, single-dose, syringes, microneedles, multi-dose, vials

## Abstract

The transition toward wide-scale use of single-dose administration systems such as prefilled syringes has primarily occurred in high-income countries due to economic considerations. This has resulted in a disparity of access to such technologies in low- and middle-income countries, which continue to utilize multi-dose vial-based presentations and syringes for parenteral delivery. Single-dose innovations currently available or in the product development pipeline represent the promise of enhanced access globally and the potential for public health impact. This perspective article discusses the reported benefits of pre-filled single-dose delivery systems compared to multi-dose vials, as well as the higher standards of infection control regulations and practices that resulted in the increasing use of and benefit from single-dose administration systems in high-income countries. We evaluated how these benefits and standards could enhance health initiatives in low- and middle-income countries. Finally, we explored the potential for making pre-filled single-dose delivery methods both accessible and affordable in low- and middle-income countries.

## 1. Introduction

The parenteral, injectable route of drug administration is a highly effective method of delivering active pharmaceutical ingredients (APIs) that have poor bioavailability. It also provides an immediate onset of action and avoids any metabolism interference associated with oral drug products. While the longstanding presentation format for injectable pharmaceutical delivery involves the combination of a multi-dose glass vial and a sterile hypodermic needle and syringe, more recent innovations have advanced the parenteral administration of drugs and vaccines. Most notably, prefilled (also known as ready-to-use (RTU)) single-dose syringes have become a preferred method of drug delivery in the past few years.

Global growth of prefilled syringes is projected to more than double by 2030, with projected annual growth rates of more than 10% [1]. In the USA and Europe, almost half of prescription-based vaccines are supplied in a prefilled syringe presentation [2]. The rising prevalence of chronic disease and growing demand for biological drugs, including self-injection applications, are major drivers of growth [3].

Until now, this trend to single-dose administration systems has, for the most part, been primarily confined to high-income countries (HICs) and related use case settings, due to economic considerations such as the high cost of existing prefilled syringe designs relative to multi-dose vials combined with needles and syringes.

Here, we provide our perspective on the reported advantages of prefilled single-dose approaches over conventional syringes and multi-dose glass vials, and the infection control standards that now limit the conditions of use for multi-dose vials in high-income countries. We examine how those advantages and standards would benefit health programs in low- and middle-income countries (LMICs). We then look at the prospects for making prefilled single-dose delivery systems accessible and affordable in resource-limited settings.

## 2. What Is Driving the Shift to Prefilled Formats in High-Income Countries?

### 2.1. Current Infection Control Standards for Use of Multi-Dose Vials

According to the United States (US) infection control standards, all medication preparation, such as filling syringes from glass vials, should occur in a dedicated clean medication preparation area (e.g., nurses’ station), away from immediate patient treatment areas. However, if there is a need to access multi-dose vials in the patient room, the vial must be dedicated for single-patient use only, the patient should be housed in a single-patient room, and all medication preparation should be performed in a designated clean area that is not adjacent to potential contamination sources (e.g., sink and used equipment) [4]. Single-dose vials or single-dose pre-packaged injection systems eliminate the need for these restrictions when giving injections.

Medications filled into multi-dose glass vials require preparing the vial by sanitizing the top with an alcohol swab and using good aseptic technique to remove a single correct dose from the multi-dose vial [5].

### 2.2. Reducing Vial-Related Dose and Contamination Errors

In newer single-dose systems, sterile content is contained within a prefilled syringe by the manufacturer. Prefilled syringes eliminate the microbial contamination risk factors associated with vial preparation and provide pre-measured, accurate doses that reduce dosing errors and save the time needed to carefully fill the syringe with a correct dose and ensure it is properly labeled, stored, and transported prior to administration [5].

The research from the US Centers for Disease Control and Prevention (CDC) presented at the Fifth Decennial International Conference on Healthcare-Associated Infections 2010 [5] showed that nosocomial infections can result from inadequate injection safety practices, and such events could be prevented by safer practices. Some of the unsafe practices were related to hygienic practices and improper storage and labeling of medications. For instance, the CDC identified as safety breaches the use of medications in multi-dose vials that were accessed multiple times with non-sterile syringes and needles. This means that bacteria, viruses, or fungi may enter the vial and cause harm to the patients who receive the drug or vaccine [5].

Published reports indicate a likely iatrogenic transmission route for many common diseases, such as Hepatitis B (HepB), Hepatitis C (HepC), and HIV, from unknowingly contaminated multi-dose vials [6]. See Table 1 for examples from HICs and LMICs.

Still, the resulting burden of disease from hundreds of millions of injections from multi-dose vials can be assumed to be significant in certain areas of the world.

Overall, the newer infection control practices with single-dose prefilled devices have brought significant reductions in preventable adverse events [7,8].

**Table 1:** Nosocomial infections from syringe misuse—HICs and LMICs.

Income Setting	Country/Setting	Year	Unsafe Practice/Outcome	Citation
HIC	Chesapeake Regional Medical Center (Chesapeake, VA Virginia, USA)	2025	Nurse reused needleless syringes for IV meds; ~330 patients notified for HBV/HCV/HIV testing.	[9]
HIC	Providence & Legacy Health (OR Oregon, USA)	2024	Potential infection-control breach by anesthesiologist; >2400 patients notified for HBV/HCV/HIV.	[10]
HIC	Los Angeles pain clinic (CA, USA)	2024	Hepatitis C outbreak linked to breaches in injection safety, multidose vials.	[11]
HIC	United States (multi-setting)	2012–2018 (published 2020)	66,748 patients notified due to unsafe injection practices (syringe/needle reuse, vial contamination).	[12]
LMIC	Ratodero/Larkana, Sindh (Pakistan)	2019–2020	Pediatric HIV outbreak; injection reuse and unsafe IV sets implicated.	[13]
LMIC	Larkana District, Pakistan	2019 (analysis 2020)	Extensive HIV outbreak attributed to unsafe injection/infusions.	[14]
LMIC	Roka commune, Battambang (Cambodia)	2014–2015	Community HIV cluster from unsafe medical injections/infusions by unlicensed provider.	[15]
LMIC	India, Unnao (Uttar Pradesh)	2018	HIV outbreak linked to unsafe injections by unqualified practitioner.	[16]
LMIC	India/Pakistan (South Asia region)	2000–2015 (review)	High prevalence of syringe reuse in therapeutic injections; outbreaks of HBV, HCV, HIV.	[17]

### 2.3. Elimination of Particulates Arising from Repeated Vial Septum Penetration Errors

One of the risks associated with vaccine delivery via glass vial containment systems is particles that can result from fragmentation and coring of rubber stoppers subjected to needle or spike punctures [18]. These rubber fragments can increase the risk of microbial contamination. Rubber particles may also cause local inflammation or trigger immune responses [19]. This particulate risk can be eliminated by using single-dose vials or prefilled single-dose systems that are contained within the injection device.

### 2.4. Reduction in Vial-Related Accidental Needlesticks

According to the U.S. Occupational Safety and Health Administration (OSHA), every year, two million healthcare professionals worldwide (out of a total of 35 million) suffer from accidental needlestick wounds [1,20]. Needlestick injury (NSI) represents the potential to transmit bloodborne disease to the healthcare worker (HCW) or those who might handle sharps in an unsafe manner. Avoiding the process of drawing out vaccines from glass vials by using ready-to-administer devices reduces the frequency of needle manipulations and thus the exposure time of the needle. These exposure reductions have resulted in lower rates of NSI [21].

### 2.5. Removal of Preservatives

Vaccines in multi-dose glass vials contain the preservative thimerosal to prevent the growth of bacteria and fungi after the vial is opened. Prefilled single-dose vaccines do not require a preservative. Despite the lack of evidence of toxic effects of thimerosal, the public perception of risk owing to this ethyl-mercury-containing preservative (as opposed to methyl-mercury),has shifted many providers in HIC away from multi-dose glass vials towards preservative-free prefilled single-dose syringes. Also, the risk of contamination of vaccines cannot be eliminated even with the use of preservatives. The literature contains several reports of bacterial contamination of vaccines despite the presence of a preservative, emphasizing the need for meticulous attention to technique in withdrawing vaccines from multi-dose vials [6,22,23].

### 2.6. Workload

Simplification of injection procedures in busy and more error-prone situations has been a major driver of change in vaccine delivery in HICs and some middle-income countries (MICs). Many studies have documented the timesaving and error prevention benefits of prefilled single-dose injection systems [24,25].

Drugs and vaccines are not always used in perfect environments. They frequently need to be deployed in emergency situations or remote areas to respond to disease outbreaks. The errors highlighted in the Institute for Safe Medication Practices (ISMP) surveys include dose or medicament mix-up due to unlabeled or incorrectly labeled syringes, incorrect dose or concentration, measurement errors, risk of drug diversion, microbial contamination, and needlestick injuries, especially when nurses compound or manipulate medications at the patient’s bedside prior to administration [8,26,27]. Single-dose pre-packaged systems are better suited to these applications due to the speed at which they can be delivered while maintaining high-level infection control and reduction in error due to the stress of the situation [8].

### 2.7. Expansion of Self-Care

COVID-19 fast-tracked the healthcare industry’s growing acceptance of patient self-injection, enabling patients to continue treatment outside of hospital environments and within outpatient facilities and home-care settings. Staff shortages, budget limits, and an aging population have contributed to the drive toward self-administration for patients with chronic disease [28]. Self-administration of injectable drug products was a growing market trend before the COVID-19 pandemic and is anticipated to continue to rise as a result of the development of novel therapeutics, biosimilars, and differentiation of existing marketed injectable products. This trend is expected to reduce the burden on healthcare systems and practitioners [29].

### 2.8. Reduction in Drug Manufacturers’ Need to Overfill

Less overfill volume is required in single-dose pre-filled injection systems (around 2% to 5%) compared to vials (typically 23% in multi-dose glass vials) [30]. Because of allowance for variable manual dose withdrawals, and the fact that the vaccine is retained in the vial by the stopper and again retained by the syringe and needle, parenteral administration from multi-dose glass vials typically requires more overfill in order to ensure that all doses of the vaccine are removed from the vial. In some circumstances, e.g., the use of a specific intradermal needle-free jet injector technology, overfill has allowed for a greater number of doses to be withdrawn from a vial, which could be beneficial at the program level [31]. More generally, however, prefilled single-dose systems can save between one and two doses of drug or vaccine per ten doses delivered when compared with ten-dose vials.

When manufacturing tens of thousands of vials of a product, this overfill volume can result in significant losses of a valuable vaccine. The virtual elimination of overfill by pre-filled single-dose systems eradicates a major source of inefficiency in the manufacturing process, allowing larger quantities of vaccines to be sold and used. This is particularly impactful for more expensive vaccines and can help to reduce costs associated with prefill presentations.

## 3. How Do Those Advantages Match with Needs and Conditions in Lower-Resource Settings?

### 3.1. Access

Reaching children or pregnant women with needed care and critical medications is an ongoing and urgent challenge. For example, global immunization coverage has stalled in the last few years, and more than 14 million children received no vaccines in 2023. An additional 6.5 million were only partially vaccinated [32]. Access requires a sufficient supply of key vaccines and drugs, but also the availability of an adequate healthcare workforce. Injection administration is generally confined to formally trained providers, a category in short supply in most low-income countries (LICs). Expanding injection capabilities to the much wider category of community health workers (CHWs) would greatly expand the workforce for the delivery of vaccines, as well as urgent medicines, such as for obstetric emergencies managed by community birth attendants.

In HICs, it is already common for routine vaccination to be presented in single-dose glass vials or prefilled single-dose syringes. In LMICs, most vaccines are contained in 10- or 5-dose glass vials. Routine immunization is often carried out in crowded single rooms with limited infrastructure, or even outdoors. Outreach to homes, schools, or outposts is common in widely dispersed populations and areas with sparse and difficult transportation links. Supplemental immunization activities (SIAs), otherwise known as immunization campaigns, are conducted in response to or in preparation for disease outbreaks. These immunization scenarios rarely allow for separate preparation stations or optimum infection control conditions. Transmission of blood-borne pathogens through injection has been a major issue for global healthcare programs [33,34].

These conditions of care are often comparable to emergency conditions in HICs and could greatly benefit from the enhanced safety and efficiency of integrated single-dose injection systems. The effectiveness of prefilled single-dose injection systems in emergency situations and the long-term experience with self-injection, which first started with morphine syrettes during the world wars, suggest that the availability of critical vaccines and drugs in prefilled unit-dose formats would greatly expand the workforce for injectables, with a significant impact on accessibility.

### 3.2. Amplification of Risk Factors in Low-Resource Settings

Some of the factors that contribute to vial contamination risk for vaccines and drugs in low-resource and low-infrastructure settings are as follows:

- Reusing needles or syringes to access the vial.
- Failing to disinfect the rubber septum before each puncture.
- Handling vials and syringes in conditions not conducive to infection control.
- Using a vial with a ruptured or disintegrated septum.

All of these factors are absent when prefilled systems are employed.

### 3.3. Wastage and Missed Opportunities

Vaccine wastage can have negative impacts on the availability, cost, and equity of healthcare programs [35]. Vaccines are routinely wasted with multi-dose glass vials when there is a mismatch between the number of doses remaining in a vial and the number of people willing or eligible to receive them prior to the time the vaccine must be discarded per the World Health Organization (WHO) multi-dose vial policy (MDVP) for safety reasons [36,37,38]. Efforts to avoid wastage can also impact access to vaccines when healthcare providers are reluctant to open a multi-dose vial for just one or two doses, and, thus, potential recipients are turned away for that day and may not be able to return—a so-called missed opportunity [39].

The overall purchase cost advantage of multi-dose vials, based upon current cost/benefit standards for vaccines, is contingent on the extent of wastage associated with their use and the cost per dose of that wastage. Cost per dose delivered is the key metric for assessing value. Assessment of wastage is critical to cost/benefit calculations, but data is not available in many countries. Where data is available, wastage varies with type of vaccine, presentation, and vial size, with lyophilized vaccines having the highest wastage rate overall [27,30,40,41].

This wastage factor has already resulted in a reduction in vial size to one or two doses for the most expensive drugs and vaccines [36]. Based upon these observations, single-dose prefilled presentations would have a very significant impact on waste reduction.

### 3.4. Vaccine Hesitancy

Vaccine hesitancy—defined by the WHO Strategic Advisory Group of Experts (SAGE) as a delay in acceptance or refusal of vaccination despite the availability of services—affects countries across all income levels. In LMICs, hesitancy interacts with access and delivery constraints; in HICs, hesitancy more often emerges as the binding constraint once services are available. Foundational frameworks such as the SAGE “3Cs” (confidence, complacency, and convenience) and the validated “5C” psychological antecedents scale are widely used to diagnose local drivers and tailor interventions [42,43,44,45].

In LMICs, hesitancy also measurably influences uptake but typically alongside supply, logistics, and service-access barriers. Multi-country studies conducted across LMICs found generally high baseline acceptance for COVID-19 vaccines—often around 70–80%—but also identified pockets of distrust and safety concerns that translated into slower uptake once supply constraints eased [46].

Systematic and narrative reviews focused on pediatric immunization similarly report that parental hesitancy—driven by confidence and convenience factors—is associated with lower completion of routine schedules (e.g., DTP and measles) in LMIC settings, reinforcing the need to pair delivery improvements with locally tailored demand-generation efforts [47]. The impact of prefilled single-dose delivery systems on vaccine hesitancy in LMICs is unclear, but they do reduce preparation steps and administration errors, improvements that directly address the “confidence” and “convenience” aspects of the WHO/SAGE 3C hesitancy framework and have been linked with increased early coverage [48,49].

## 4. What Is Preventing Uptake of Pre-Filled Single-Dose Delivery Systems in Lower Resource Settings?

### 4.1. The Progression of Injection Practices for Vaccination in Low- and Middle-Income Countries

Risks associated with syringe reuse and associated vial contamination in LMIC came into critical focus in the late 1970s when efforts by the new WHO Expanded Program on Immunization (EPI) were gaining momentum. For tackling the challenges of reaching all of the world’s children with vaccines, injections needed to become accessible for communities with harsh climates, little support, and few resources. Glass reusable syringes were in use at the time, decontaminated by boiling for five minutes.

Boiling does not achieve sterilization, and boiling temperatures or durations were, in any case, frequently not achieved. Steam sterilization via adapted pressure cookers was also introduced, but adequate heat sources were often unavailable. Multiple studies have documented links between unsafe injection and the transmission of HepB and HepC, HIV, Ebola, and Lassa Fever [33].

EPI moved swiftly to change practice, subsequently moving to plastic disposable syringes. These low-cost devices, intended for single use, were responsible for largely eliminating syringe reuse in high-income countries. However, in resource-poor settings, objects of perceived value are rarely thrown away if they remain functional.

Continued re-use of disposable syringes prompted EPI to call for engineered solutions. In the 1990s, fixed and variable dose auto-disable (AD) and re-use prevention (RUP) single-use syringes were introduced into immunization programs. These devices, along with other developments to monitor heat exposure, helped to mitigate disease transmission, vaccine vial contamination, or spoilage. However, risks associated with failures in aseptic vial handling technique, vaccine wastage, rubber particulates from the septum, glass delamination, and dosing errors under challenging conditions of care remain for multi-dose vials. Also, while vaccine syringes from the United Nations Children’s Fund (UNICEF) have been required to be auto-disabled since 2000, AD syringes represent less than 10% of the syringe injections per year in LMIC [50].

### 4.2. Planning for Improvements

Because of those enduring risks, the prospect of single-dose contained injection systems has high appeal for global healthcare programs. In 2000, the WHO, the United States Agency for International Development (USAID), UNICEF, and collaborators issued a report entitled “Technologies for Vaccine Delivery in the 21st Century” [51]. In their summary, they state the following: “We can now envision a vaccine delivery system that utilizes safe prefilled injection devices containing single doses of thermostable vaccine.” They viewed this development as critical to achieving equity in access to new vaccines, safety of vaccine administration, and simplicity and efficiency of vaccine delivery.

WHO best practice guidelines for infection control recommend the importance of using single-dose rather than multi-dose vials whenever possible [23,52]. While use of single-dose containers in resource-limited environments has been limited due to financial resource and supply system constraints, the burden of maintaining infection control standards and cost efficiency with multi-dose vials falls primarily to healthcare workers [53]. Best practice recommendations include preparing each injection in a clean designated area, performing hand hygiene or washing soiled skin before preparing injection material, and inspecting medication for breaches of integrity, and all are conditioned by the circumstances prevailing at the point of care, which are often sub-optimal in low-resource settings.

In the face of continued unsafe injection practices, in 2015, WHO called for the worldwide use of “smart syringes” to curb infections from unsafe injections [54]. In 2016, this statement was followed by a WHO guideline on the use of safety-engineered syringes in healthcare settings [55]. In a more recent exercise by global health agencies and partners (Vaccine Innovation Prioritization Strategy, [VIPS]), two single-dose innovations, microarray patches (MAPs), also known as microneedle array patches, and compact prefilled auto-disable (CPAD) device concepts were ranked among the top nine priorities out of twenty-four innovations considered, with MAPs in the top three [56].

In response to the recent WHO reports on the slowing of vaccine coverage worldwide [56], WHO states that “Vaccine product innovations that ease delivery in resource-constrained settings are urgently needed to achieve the Immunization Agenda 2030 (IA2030) goals on equitable vaccination coverage.” [57].

Although current forms of prefilled syringes available in the HIC would address these problems, they are not affordable for resource-limited healthcare programs in LMICs and communities. They are complex to manufacture, requiring separate steps to mold and sterilize components and fill in-line under sterile conditions. Global health leaders are seeking alternative single-dose prefilled delivery systems for vaccines and drugs that can be produced at high scale and low cost with rapid, simple, and reliable manufacturing processes and that are minimally disruptive to current practice.

LMIC populations continue to experience major inequities due to the lack of access to affordable single-dose vaccines and drugs, delivery system challenges, and the consequent reliance on multi-dose vials under conditions that do not comply with current infection control standards. Emerging technologies aim to overcome most of the barriers that currently prevent these programs from gaining the safety and efficiency advantages available in high-income countries.

## 5. New Prospects for Cost-Effective Single-Dose Delivery Systems in Global Health

Two main forms of alternative delivery technologies are in advanced development: (1) compact prefilled devices, most with a form based upon the original syrette device used to self-deliver morphine to soldiers in the great wars of the twentieth century; and (2) microarray patches (MAPs), a new format than allows vaccines or drugs in solid form to be administered directly into the skin.

### 5.1. Compact Prefilled Injection Devices

#### 5.1.1. Form–Fill–Seal Injectors

Form–fill–seal devices were the first prefilled single-dose injection devices to be used in global health programs. The advantages of prefilled single-dose vaccine and drug delivery were recognized by the EPI in the mid-1980s. This led to the development and introduction of a new type of prefilled injection system in the form of an injection-molded squeezable blister with a one-way valve (to prevent reuse—an AD feature) and fixed needle: the compact prefilled auto-disable (CPAD) device technology with the commercial name BD Uniject™ (https://www.bd.com/en-us, accessed on 6 April 2025). It is molded, terminally sterilized, and then delivered to the pharmaceutical company, where it is filled and heat-sealed under sterile conditions. Uniject™ obtained regulatory clearance and passed WHO product quality standards in the early to mid-1990s. The device assures sterility of the dose up to the point of delivery into the body, prevents reuse through the AD feature, and generates 30% less waste than single-dose vials and standard syringes [58].

Interest in Uniject™ led to multiple field evaluations and studies for vaccines such as HepB [59,60,61,62,63], tetanus toxoid [64], and pentavalent vaccine [65], as well as drugs such as gentamicin [66,67] for neonatal sepsis, or for women’s health, such as oxytocin [68,69,70,71,72,73,74], Cyclofem [75,76], and Depo-Provera [77,78,79,80,81,82]. Several pharmaceutical companies initially took up the product for HepB, tetanus toxoid, and pentavalent vaccines. Others explored the use of the format for injectable contraceptives. The use of this prefilled device proved highly acceptable to healthcare providers and their patients [83]. Individuals who had never delivered an injection were able to do so after minimal training, and the CPAD format was preferred over standard needle and syringe [84].

Using this system, UNICEF was able to deliver nine million doses of tetanus toxoid to women in remote populations throughout the world, including Afghanistan, Ghana, and Malawi [85]. Also, experience with Uniject™ prefilled single-dose injection systems for the birth dose of HepB vaccine in Indonesia has demonstrated the potential for prefilled single-doses to effect policy change, allowing birth attendants to deliver birth-dose vaccines [86]. This opens the opportunity for CHWs worldwide to be allowed to administer prefilled single-dose injections with country-specific regulatory approval.

The Uniject™ device is still available for uptake by pharmaceutical companies. However, nearly three decades after its introduction, Uniject™ uptake in LMIC has been limited to only three applications—for the birth dose of the HepB vaccine in Indonesia [86]; historically for tetanus toxoid in the same region, although it is now discontinued as a product; and, more broadly, for subcutaneous injectable contraceptives. In particular, there have been expanded investments and commitment to Uniject™ for contraceptives, with an emphasis on self-administration [87,88].

Uniject™ has demonstrated high acceptability and public health impact of compact prefilled delivery devices in LMIC. However, there are lessons to be learned about factors that limit uptake of combination single-dose delivery products for resource-limited markets, since the CPAD Uniject™ was found highly acceptable by healthcare providers and patients and has been used to deliver HepB vaccine in limited markets and used more broadly in injectable contraceptive delivery for the last two decades. However, it failed to scale more broadly. Closer examination of this pioneering prefilled device may help to address outstanding stakeholder issues with new designs and enable resource-poor communities to gain the benefits of single-dose prefilled injection systems. Several factors may have contributed to this outcome. For global health programs, the cost of Uniject™ is similar to the cost of a standard syringe and single-dose vial, but significantly higher than the per-dose procurement cost of multi-dose vials with standard syringes [89]. This cost premium limited uptake to special applications such as the injection of higher cost APIs.

#### 5.1.2. Blow–Fill–Seal Injectors

Blow–fill–seal (BFS) is an established and industry-recognized advanced aseptic fill–finish process for sterile liquids where the pharmaceutical-grade plastic container is formed from liquid plastic, immediately filled with the liquid drug product, and then sealed in a rapid, continuous sterile process. The BFS manufacturing process creates, fills, and seals the drug container in one continuous aseptic process that takes seconds. A single BFS production line can manufacture up to 15 million prefilled units per month. It is globally accepted by regulatory authorities and used around the world to package billions of doses of both large and small molecule sterile drug products annually, including oral vaccines [90].

The advantages of BFS for sterile containers are as follows:

- Very efficient manufacturing process with almost no human intervention.
- Commercially available and rapidly scalable basic manufacturing plant.
- Aseptic filling in a closed International Organization for Standardization (ISO) Level 5 environment.
- Single primary raw material: pharmaceutical-grade polymer resin.
- Lightweight and resistant to breakage.
- Low-cost unit-dose filling.
- Highly customizable container shapes and volumes.

Blow–fill–seal (BFS) containers for medical and pharmaceutical applications are widely used because of their core properties:

- Flexibility.
- Squeezability.
- Clarity.
- Assured sterility.
- Lightweight and shatterproof.
- Highest quality combined with lowest cost.
- Significant environmental advantage over glass vials and standard syringes.

Although BFS containers appear ideal for delivery of small-volume parenteral (SVP) pharmaceuticals, injectable versions have not emerged until recent years [91]. More recently, robust designs have been developed targeting the needs for rapid scale-up in response to pandemics as well as for more affordable prefilled single-dose delivery systems for LMIC health programs. These devices combine the finished container with a proprietary, insulin-style needle hub to create a high-quality, RTU injection device. BFS combines the efficiency and low cost of high-throughput production with the highest guarantee of sterility and adaptability to a variety of container sizes and configurations. The entire process is supported by a simple supply chain that relies on three readily available and widely used raw materials—low-density polyethylene (LDPE) plastic and polypropylene resin, as well as stainless-steel cannula—all of which can be stockpiled.

##### Advantages

The key advantages to BFS injectables are a flexible, rapid device design process that can meet many pharmaceutical product needs; an affordable prefilled format; a simple supply chain; a giant production scale; short lead times; a simple, easy-to-use design that opens the door to self-administration or use by CHWs in LMICs; and a lower carbon footprint by avoiding glass and using less plastic than a traditional syringe. In particular, an environmental impact study for a specific single-dose BFS injectable design indicated a 65–125% lower carbon footprint per dose compared to multi-dose and single-dose vials and Luer-type prefilled syringes. Additionally, the BFS design used approximately 100 times less water in manufacturing compared to single-dose vials [92]. BFS-based injection systems incorporating AD features have the potential to mitigate the cost and logistical obstacles to widespread use of single-dose prefilled syringes in LMIC populations and can have multiple benefits, potentially making them a highly desirable option for vaccine and drug delivery in these regions [89]. Although commercial BFS-based injection systems are not yet available, early predictions (Sedita et al. 2018) [89] of delivery cost per dose compared to existing formats project a significant (15–20%) reduction in cost compared to single-dose glass vials and available preformed CPAD (Uniject™). Ten-dose glass vials yielded the lowest cost in this study due mainly to the cost of transportation and storage, suggesting that although BFS-based injection systems incorporating AD features have the potential to mitigate the cost and logistical obstacles to widespread use of single-dose prefilled syringes in LMIC populations and can have multiple benefits, they are likely to carry a cost premium over current MDV standards [89].

##### Challenges

In order to gain these benefits, adjustment to the vaccine cold chain would be necessary in some settings to accommodate single-dose formats. Restructuring of vaccine supply chains for the 21st century has been a major focus in the last two decades to increase the efficiency and effectiveness of the cold chain and to accommodate the doubling of antigens and several new vaccine schedules needed to accommodate them [85,86,93,94,95]. While replacing multidose vials with CPAD primary containers is likely to significantly reduce wastage, resulting in some gain of cold chain capacity, CPAD designs can be as compact as single-dose glass vials [89]. However, higher cold chain volumes would still need to be accommodated.

Vaccine vial monitors (VVMs) are added to vaccine containers to provide a visual indication of heat exposure, which can damage the vaccine. Single-dose containers multiply the VVM cost per dose when compared with multi-dose vials. With compact BFS containers, this cost may be mitigated by the design of multi-dose dispensing systems that conserve a single VVM to protect all remaining doses, such as GSK’s oral multi-monodose ROTARIX vaccine in BFS format [96].

The following are examples of BFS injector developers.

**Figure 1:**
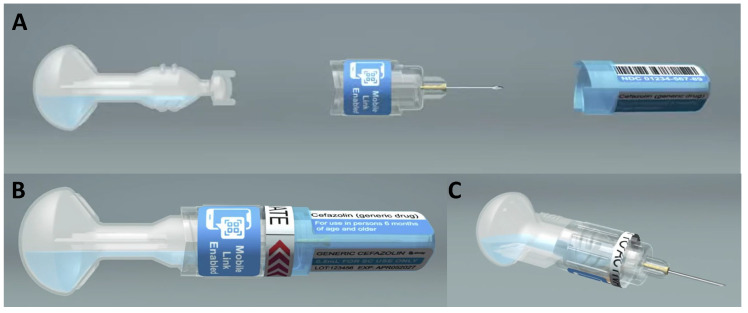
ApiJect design for LMIC use. (**A**) Pre-assembly, (**B**) assembled device, and (**C**) device ready for injection.

ApiJect Systems Corporation, Stamford, CT, USA (https://www.apiject.com/, accessed on 6 April 2025): ApiJect Ltd. was formed in the United Kingdom in 2015, and became ApiJect Systems, a US Public Benefit Corporation, in 2018. In 2019, when the US government signed an agreement with the company to begin development of emergency response capacity, it was before the emergence of COVID-19. When COVID-19 emerged months later, ApiJect intensified its focus, with additional support from the US government, on creating a device and sterile fill–finish process that could be manufactured at a population scale [97,98,99]. In September 2025, the company filed for regulatory clearance for its first prefilled BFS injection device, which is suitable for sterile liquid doses of 1.0 mL or less and intramuscular administration [100]. ApiJect has a technology development center in Orlando (FL, USA) to accelerate production engineering with a full in-house product development team that takes prototypes through all the design and development steps needed to manufacture at scale. Additionally, Tae-Chang Industrial (Gongju-si, Republic of Korea) is a partner for needle hub development and supply. ApiJect devices are manufactured using Rommelag (Sulzbach-Laufen, Germany, https://www.rommelag.com, accessed on 6 April 2025) machines; the two companies work in close partnership [101]. Concurrent with this effort, the development of designs suitable for use in LMICs for vaccination or drug delivery, as well as automated assembly systems, was also advanced through ApiLabs, with their concept design studio in London (UK). See Figure 1.

Recent innovations have demonstrated the feasibility and scalability of a novel design for BFS-based CPADs, the category of prefilled injection systems generally required for injection in low- and middle-income countries. The Gates Foundation has supported ApiJect for the development of this low-cost BFS CPAD suitable for the delivery of essential medicines and vaccines in LMICs [102]. Recent user trials in India have shown high acceptability and low training burden among volunteer injectable contraceptive users [103].

Euroject (https://www.unither-pharma.com/innovation/euroject/, accessed on 6 April 2025): Unither Pharmaceuticals–Paris, France (a contract development and manufacturing (CDMO) Pharmaceutical Subcontractor—Unither Pharmaceuticals (https://www.unither-pharma.com/, accessed on 6 April 2025) has also developed a BFS RTU syringe called Euroject™, which received funding from the French government to support manufacturing during the COVID-19 pandemic and committed 68 million EUR to advance their technology for manufacturing scale [104]. The Euroject™ design has two main components separated for shipment—the BFS container and the needle hub, which has the Luer connector, a needle, and a needle cap with a potential safety lock option. LDPE is the material utilized, and the dose volume is 0.3 mL to 0.5 mL. Per Unither, any type of needle can be used with the Euroject™ [105]. The BFS container is opened, and the needle hub is then attached to allow for dose delivery. Unither has stated that they have a dedicated Biosafety Level 2 (BSL2) facility in Amiens, France, with a capacity of up to 1 billion doses per year, and with the use of a high-speed rotary machine [106].

Brevetti Angela (Arzignano, Italy, https://www.brevettiangela.com/, accessed on 6 April 2025): Brevetti Angela (Blow–Fill–Seal Technology for Aseptic Packaging—Brevetti Angela) is an Italian BFS technology developer of both fill–finish equipment and proprietary designs for SVP and large-volume parenteral (LVP) applications. The company was founded in 1977, shifted into the pharmaceutical application area in the 1980s, and released the SYFPAC^®^ in 1989. In 1999, Brevetti Angela introduced the first BFS machine for the production of prefilled syringes. In 2004, the company marketed the SYFPAC^®^ SECUREJECT^®^ prefilled syringe, followed by the release of the SYFPAC^®^ SVP TWIN, which, according to the company, increased production by 60%. SYFPAC^®^ SECUREJECT^®^ can comprise different polyolefins (polypropylene, polyethylene, high-density polyethylene) and is capable of a volume range from 0.5 to 20 mL [107]. Brevetti Angela has also developed a BFS CPAD design, created by their spin-off company 3CK Medical Devices (Arzignano, Italy, http://3ckmed.com/, accessed on 6 April 2025), utilizing the SYFPAC^®^ BFS as a design basis. The design is informed by a CPAD target product profile published by PATH [108]. The design is intended to be pre-assembled and RTU, with a prefilled volume of 0.50 mL for vaccines for administration by HCW or 0.65 mL for contraceptive delivery and potentially self-administration with a one-way valve that serves as the RUP feature. The needle is assembled in the BFS process through an insert loaded through an isolator, with upwards of 5000 to 20,000 units per hour in manufacturing capacity [109].

### 5.2. Micronarray Patches (MAPs)

Microarray patch (MAP) technology has been in development for over the past two decades or more. Currently, there are nearly 100 public and private sector developers focused on developing the technology platform for a variety of drugs and biologics, targeted for use in HIC and LMIC markets [110]. MAP platforms can come in different formats, be it a completely dissolvable microneedle matrix containing the active pharmaceutical ingredient, a solid substrate that is coated with the active, or other formats such as hollow microneedles or porous arrays to allow for drug delivery. Some MAP technologies have demonstrated improved thermostability compared to a vial-based presentation, which could enable the distribution and use of the technology outside the cold chain. Antigen reduction and reduced need for an adjuvant have also been demonstrated with the technology platform [111].

As noted previously, MAP technology has been prioritized by global stakeholders through the VIPS initiative, which has developed a product-specific action plan for the technology class [112]. From a global public health perspective, measles–rubella MAPs (MR-MAPs) have been a focus of donor investments, with a target product profile being developed through WHO and UNICEF collaboration and through engagement with several global public health partner organizations [113,114].

Recent clinical trial results have been reported by Micron Biomedical (Atlanta, GA, USA, https://www.micronbiomedical.com/, accessed on 6 April 2025) (Phase I/II) [115] and Vaxxas Pty (Vaxxas :: Home, Hamilton, QLD, Australia, https://www.vaxxas.com/, accessed on 6 April 2025) (Phase I) [116] for MR-MAPs; these studies were funded by the Bill & Melinda Gates Foundation. Both studies reported positive results, and currently, these developers are each preparing for a full Phase II study, which will be utilized to validate product performance prior to embarking upon a pivotal safety and effectiveness Phase III study and other necessary descriptive studies, which, if successful, can ultimately lead to regulatory submission, review, product approval, and WHO prequalification. A global meeting was convened in New Delhi in April 2024 by WHO, which is advancing both the design of the Phase III trial, which has recently been published [117], and also considerations for WHO policy [118,119].

The Biomedical Advanced Research and Development Authority (BARDA) has provided multiple investments in MAP technology for epidemic and pandemic response [120,121]. The Coalition for Epidemic Preparedness Innovations (CEPI) is a global-level foundation that has also invested in MAP technology [122].

#### 5.2.1. Advantages

MAP technology offers multiple potential benefits that could address the issues of injection safety, vaccine supply chain challenges, vaccine hesitancy, as well as improved ease of use for HCWs and the prospect of self-administration (already demonstrated in the case of cosmetic MAPs that can be purchased off the shelf) [123]. It could be a critically important technology to improve immunization coverage and help achieve measles–rubella (MR) elimination. Other vaccine or essential medicine application areas could benefit from the technology as well. Global public health stakeholders have identified priority viruses for MAP technology from 91 potential vaccines. These viruses include HepB, MR/measles–mumps–rubella, human papillomavirus, rabies, yellow fever, influenza (seasonal and pandemic), and SARS-CoV-2 (priority group 1). Vaccines against group B streptococcus, Neisseria meningitidis A,C,W,Y,(X), Salmonella Typhi, and Streptococcus pneumoniae were reported to be in priority group 2 [124].

#### 5.2.2. Challenges

The potential of vaccine MAP technology and its probable influence on worldwide health is quite convincing; nevertheless, the current task is to close the distance between initial clinical development and business viability. Multiple areas present challenges, including vaccine or drug applications and the necessary clinical research for product realization, scaling up manufacturing, and navigating regulatory and funding aspects. All MAP vaccine clinical studies published so far have used vaccines that are already available as an injectable product, leveraging existing safety information, immunogenicity data, and manufacturing quality. To be successful, MAP products will require large-scale automated manufacturing using advanced microfabrication methods, reliable dosing controls, and quality assessment systems. Vaccine MAP developers face new challenges in terms of manufacturing processes, capabilities, and infrastructure. Advanced trial manufacturing runs will be necessary to convince key players in the vaccine industry that a new vaccine MAP platform will be cost-effective for large-scale production compared to existing methods. However, the business case for developing an existing vaccine with a new delivery technology needs thorough evaluation, considering the need for repeating clinical studies, the complexity, and the cost, which vary depending on the vaccine and the expected benefits of MAP delivery.

One key challenge for MAP technology is the application of the platform to other drugs and vaccines—each application represents its own product development pathway, given the requirement of reformulation for the API to be compatible with the MAP format. Such development efforts require substantial investments to advance such products forward, thus making it necessary that commercial value (ROI) be evident compared to existing product formats, or that such efforts are de-risked through public sector/donor investment. Developing a MAP delivering a vaccine that is not yet approved reduces or eliminates the need for this repetition, but comes with the risk that the vaccine’s effectiveness has not been demonstrated yet. The regulatory pathway for the technology class as it relates to vaccination and global public sector use also needs to be elucidated [125].

#### 5.2.3. MAP Developers

Micron Biomedical: The Micron Biomedical measles–rubella vaccine microneedle patch (MRV-MNP) is a solid dissolvable MAP without an applicator. An integrated button feature allows for both application to the skin and a visual cue that the MAP has been successfully delivered. The company completed a Phase I/II age-de-escalation trial in cooperation with researchers from the London School of Hygiene and Tropical Medicine Medical Research Council Unit, The Gambia. The study was a double-blind, double-dummy clinical trial to assess the safety and immunogenicity of the MRV-MNP. The trial involved 45 adults, 120 toddlers, and 120 infants. The results showed that the MRV-MNP vaccine was well-tolerated and induced strong immune responses against measles and rubella [115,126]. The company is preparing for a Phase II study with PATH and is working on automating the manufacturing process for future trials and commercial products [127].

Vaxxas Pty: The Vaxxas Pty. Ltd. (Vaxxas) platform is a high-density microarray patch (HD-MAP) with an applicator. The patch contains thousands of projections on a square centimeter area and is coated with a liquid vaccine using a non-contact printing method. The company conducted a Phase I study for MR in previously vaccinated, healthy young adults. The study demonstrated safe, tolerable delivery of a single high and low dose of MR vaccine by Vaxxas’ HD-MAP product applied for 60 s. Immune responses were similar to subcutaneous needle and syringe comparator at low and high doses; responses were related to incoming immunological status (seropositive—prior immunization) [116]. They also plan to conduct a Phase I/II MR-MAP age de-escalation study (adults, toddlers, and infants) similar to the Micron study conducted in The Gambia [128,129].

### 5.3. Summary

Emerging single-dose delivery systems for global health are advancing through two main technology pathways: CPADs and MAPs. CPADs evolved from the Uniject™ design, first introduced in the 1990s, to simplify vaccine and drug administration while preventing reuse. Though highly acceptable among users and instrumental in programs such as birth-dose Hepatitis B and injectable contraceptives, Uniject™ faced limited scale-up due to cost constraints compared to multi-dose vials. A newer generation of prefilled systems using BFS technology—developed by firms such as ApiJect, Unither (Euroject™), and Brevetti Angela—combines aseptic, high-speed manufacturing with lower material and environmental costs, potentially enabling large-scale, affordable sterile injectables for LMICs. Meanwhile, MAP technologies—solid or coated microneedle arrays that deliver vaccines or drugs directly into the skin—are progressing toward regulatory approval with promising Phase I/II trial results from Micron Biomedical and Vaxxas for measles–rubella vaccines. Backed by CEPI, BARDA, WHO, UNICEF, and the Gates Foundation, MAPs offer major advantages in thermostability, ease of use, safety, and potential for self-administration, though large-scale manufacturing, cost-effectiveness, and regulatory pathways remain key challenges to achieving widespread adoption. See Table 2 for a comparison of the technologies discussed in this perspective paper.

## 6. Discussion

Although limited to SE Asia for vaccine delivery and to injectable contraceptives in other countries, WHO prequalification (PQ) and three decades of experience with a prefilled single-dose delivery system have demonstrated their potential in global public health to enhance safety, reliability, and equitable access to vaccines and essential medicines [130]. For LMICs, these systems can mitigate risks associated with multi-dose vials—including contamination, wastage, and dosing errors—while also simplifying logistics and enabling administration by community health workers [87,130]. However, while currently available CPAD devices have been shown to be highly acceptable to users and beneficiaries [70], the limited uptake underscores the dependence of advantageous new tools in global health upon affordability [89]. This includes the innovations’ at-scale cost per-use and associated costs of introduction, such as training, logistics, and changes in cost to each sector in the supply chain [89]. The advantages of these emerging systems are indisputable, but the cost factors remain to be established following uptake by drug and vaccine suppliers relevant to the global health communities.

Each system has unique benefits that can be applied to different vaccine or drug formats. MAPS can deliver dry (and potentially heat-stable) vaccines directly into the dermis without any liquid phase [124]. With this technology, manual reconstitution of lyophilized vaccines could be eliminated, and cold chain requirements could be significantly reduced.

The well-known production efficiencies of BFS containers [131,132] coupled with novel hub and needle systems can result in lower-cost combination parenteral products. If available with a range of priority injectable drugs and vaccines, these more affordable prefilled unit-dose injection systems could greatly expand the uptake of CPAD devices in LIC and MIC settings. Many pharmaceutical products, including oral vaccines, already exist in BFS containers [131], lowering the barriers to commercial adoption of BFS injectable systems. Since these prefilled products are likely to be highly price-competitive in some HIC markets, the prospects for commercial uptake are good [89]. Furthermore, the flexibility of current state-of-the-art BFS systems to switch efficiently between different drugs or vaccines [131] changes the value proposition for addressing the needs of LIC markets.

Independent case studies or economic evaluations conducted in various LMIC settings will be required to validate and prioritize these innovations for global health markets [133,134]. However, as outlined in this document, commercial and public commitment to these developments is sufficiently advanced to suggest that the value proposition offered by these technologies will be thoroughly evaluated and product uptake facilitated according to the outcome. Products based upon these two systems offer the best chance of reducing dependence on multi-dose vials in resource-poor settings.

## 7. Conclusions

This perspective highlights both the opportunities and limitations of prefilled single-dose systems compared to multi-dose vials. While infection control, dosing accuracy, and reduced wastage clearly favor prefilled devices, challenges remain in achieving affordability and cold chain adaptation. However, recent progress has increased the prospect of achieving affordable pre-filled systems in the next five years.

## 8. Future Directions

To enable the advancement of vaccine and drug delivery standards of care in LMIC health programs, equivalent to those in high-income countries, future efforts should

1. Encourage evaluation and uptake of these cutting-edge technologies by pharmaceutical and fill and finish companies.
2. Conduct cost-effectiveness and implementation pilots in LMIC health programs.
3. Global public health stakeholders should encourage and support international and local BFS manufacturing at scale for single-dose presentations of priority drugs and vaccines to reduce per-dose costs while maintaining quality and regulatory compliance.
4. Global public health stakeholders should accelerate the development of MAP technologies for multiple vaccines to enable thermostable, needle-free vaccination.
5. Incorporate vaccine hesitancy, workforce limitations, and behavioral insights into technology adoption strategies.

## Figures and Tables

**Table 2:** Comparison of emerging single-dose delivery technologies for global health applications.

Feature	Compact Prefilled Auto-Disable (CPAD)	Blow–Fill–Seal (BFS) Injectables	Microarray Patches (MAPs)
Representative Products/Developers	BD Uniject™; PATH legacy programs (vaccines and drugs)	ApiJect Systems; Unither Pharma (Euroject™); Brevetti Angela/3CK Medical	Micron Biomedical; Vaxxas Pty Ltd.
Primary Material/Construction	Injection-molded polymer blister with fixed needle and one-way valve	LDPE or PP plastic formed, filled, and sealed aseptically in one step	Solid or coated microneedle arrays (polymer, silicon, metal, or sugar matrices)
Dose Format	Prefilled liquid single dose	Prefilled liquid single dose	Solid or coated dry-state dose
Sterility Assurance	Terminal sterilization before filling; sterile fill–seal	Closed-loop aseptic BFS process (ISO 5)	Intrinsically sterile solid micro-projections; no liquid phase
Auto-Disable/Re-Use Prevention	Built-in one-way valve prevents re-use	Optional integrated AD hub in design prototypes	No needle—eliminates reuse risk
Cold-Chain Requirements	Similar to liquid injectables	Similar to liquid injectables; potential for lower energy footprint	Reduced or eliminated for thermostable MAPs
Manufacturing Scalability	Proven small- to medium-scale; high setup cost	Extremely high throughput (up to 15 million units/month per line)	Limited; automation and scale-up in development
Unit-Dose Cost (Projected vs. MDV)	~equal to single-dose vial + syringe; higher than MDV	15–20% lower than single-dose glass vials; likely > MDV [89]	TBD—currently higher; expected to decline with scale
Environmental Impact	~30% less waste than vial + syringe	65–125% lower CO_2_ footprint; 100× less water use [89]	Minimal sharps or packaging waste
Regulatory/WHO PQ Status	WHO prequalified for HepB birth dose and tetanus toxoid (Indonesia) and pentavalent vaccines; used for contraceptives, including approval for self-administration	Under regulatory review (e.g., ApiJect 2025); BFS widely accepted for sterile packaging	WHO/UNICEF TPP in development; Phase II–III trials for MR-MAP
Key Advantages	Proven field use; easy training; CHW-ready	Rapid, scalable aseptic manufacturing; lower carbon footprint; customizable	Needle-free; potential thermostability; minimal training; reduced hesitancy
Key Challenges	Cost premium over MDV; limited uptake	Cold-chain capacity; regulatory adaptation for injectables	Manufacturing scale-up; regulatory pathway; reformulation needs
Ideal Use Cases	Vaccines or drugs ≤ 1 mL; CHW delivery; self-administration	Pandemic response; mass immunization; low-cost LMIC fill–finish	Thermostable vaccines; outreach campaigns; self-use and pediatric delivery

## Data Availability

No new data were created or analyzed in this study. Data sharing is not applicable to this article.

## Abbreviations

The following abbreviations are used in this manuscript:

AD	Auto-disable
API	Active pharmaceutical ingredient
BARDA	Biomedical Advanced Research and Development Authority
BD	Becton, Dickinson & Company
BFS	Blow–fill–seal
BSL2	Biosafety Level 2
CDMO	Contract development and manufacturing organization
CEPI	Coalition for Epidemic Preparedness Innovations
COVID-19	Coronavirus disease
GSK	GlaxoSmithKline
CHWs	Community health workers
CPAD	Compact prefilled auto-disable
EPI	Expanded Program on Immunization
HCW	Healthcare worker
HepA	Hepatitis A
HepB	Hepatitis B
HepC	Hepatitis C
HD-MAP	High-density microarray patch
HIC	High-income countries
HIV	Human immunodeficiency virus
IA2030	Immunization Agenda 2030
ISMP	Institute for Safe Medication Practices
ISO	International Organization for Standardization
LDPE	Low-density polyethylene
LICs	Low-income countries
LMICs	low- and middle-income countries
LVP	Large-volume parenteral
MR-MAPs	Measles–rubella MAPs
MAPs	Microarray patches
MICs	Middle-income countries
MDVP	Multi-dose vial policy
MNP	Microneedle patch
MRV	Measles–rubella vaccine
NSI	Needlestick injury
ROI	Return on investment
RTU	Ready-to-use
RUP	Re-use Prevention Feature
SARS	Severe acute respiratory syndrome
SVP	Small-volume parenteral
SIAs	Supplemental immunization activities
OSHA	U.S. Occupational Safety and Health Administration
UK	United Kingdom
UNICEF	United Nations Children’s Fund
US	United States
USAID	United States Agency for International Development
CDC	US Centers for Disease Control and Prevention
VIPS	Vaccine Innovation Prioritization Strategy
VVMs	Vaccine vial monitors
WHO	World Health Organization
